# Expression of Novel *L*-Leucine Dehydrogenase and High-Level Production of *L*-Tert-Leucine Catalyzed by Engineered *Escherichia coli*

**DOI:** 10.3389/fbioe.2021.655522

**Published:** 2021-03-30

**Authors:** Yuan-Yuan Jia, Yu-Li Xie, Lu-Lu Yang, Hong-Ling Shi, Yun-Feng Lu, Si-Pu Zhang, Cun-Duo Tang, Lun-Guang Yao, Yun-Chao Kan

**Affiliations:** ^1^Henan Provincial Engineering Laboratory of Insect Bio-reactor, Henan Key Laboratory of Ecological Security for Water Source Region of Mid-line of South-to-North, Nanyang Normal University, Nanyang, China; ^2^School of Life Sciences and Agricultural Engineering, Nanyang Normal University, Nanyang, China; ^3^Henan Academy of Agricultural Sciences, Zhengzhou, China

**Keywords:** leucine dehydrogenase, feed additive, expression, biocatalysis, formate dehydrogenase

## Abstract

Leucine dehydrogenase (LDH) is a NAD^+^-dependent oxidoreductase, which can selectively catalyze α-keto acids to obtain α-amino acids and their derivatives. It plays a key role in the biosynthesis of *L*-tert-leucine (*L*-Tle). As a non-naturally chiral amino acid, *L*-Tle can be used as an animal feed additive, nutrition fortifier, which is a perspective and important building block in the pharmaceutical, cosmetic, and food additive industry. In this study, four hypothetical leucine dehydrogenases were discovered by using genome mining technology, using the highly active leucine dehydrogenase *Ls*LeuDH as a probe. These four leucine dehydrogenases were expressed in *Escherichia coli* BL21(DE3), respectively, and purified to homogeneity and characterized. Compared with the other enzymes, the specific activity of *Pf*LeuDH also shows stronger advantage. In addition, the highly selective biosynthesis of *L*-Tle from trimethylpyruvic acid (TMP) was successfully carried out by whole-cell catalysis using engineered *E. coli* cells as biocatalyst, which can efficiently coexpress leucine dehydrogenase and formate dehydrogenase. One hundred-millimolar TMP was catalyzed for 25 h, and the yield and space-time yield of *L*-Tle reached 87.38% (*e.e.* >99.99%) and 10.90 g L^–1^ day^–1^. In short, this research has initially achieved the biosynthesis of *L*-Tle, laying a solid foundation for the realization of low-cost and large-scale biosynthesis of *L*-Tle.

## Graphical Abstract

Genome mining of Leucine dehydrogenase and one-pot synthesis of *L*-tert-leucine from trimethylpyruvic acid.

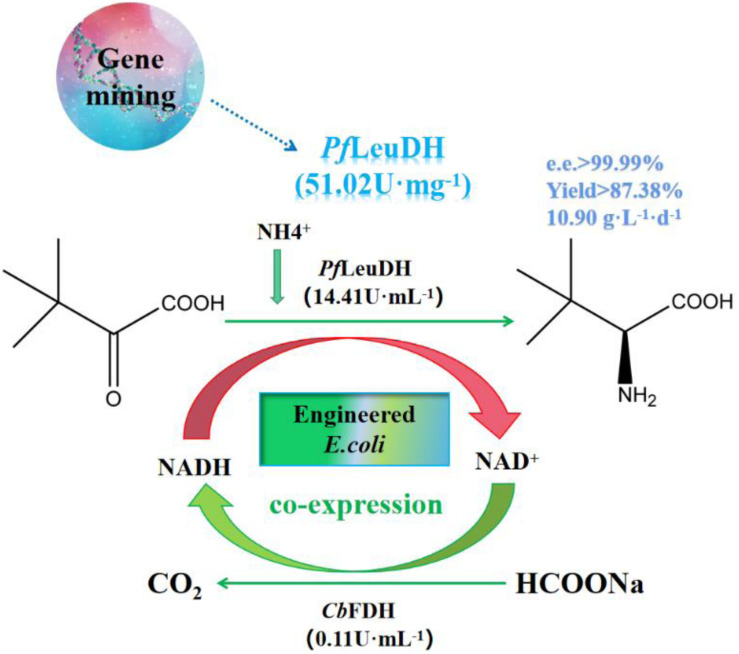

## Introduction

With the rapid development of synthesis technology, unnatural amino acids have been widely used in many fields, mainly covering the food, pharmaceutical, and chemical industries (Luo et al., 2020). Due to the unique chemical and biological properties of unnatural amino acids, many unnatural amino acids are widely used as chiral drug intermediates in the pharmaceutical field to produce synthetic chiral drugs (Blaskovich, 2016; Almahboub et al., 2018; Narancic et al., 2019; Ouyang et al., 2020). *L*-Tert-leucine (*L*-Tle) is a non-naturally chiral amino acid whose tert-butyl group has significant hydrophobicity and steric hindrance. It can effectively control the conformation of polypeptide molecules in polypeptide biosynthesis to form chiral compounds (Bommarius et al., 1995; Wang et al., 2020). *L*-Tle can be used as an animal feed additive and a nutritional fortifier in the food and cosmetic fields (Jin et al., 2011). In addition, it is also an intermediate for the synthesis of biological inhibitors and antiviral drugs, such as HIV protease inhibitors and hepatitis virus inhibitors (Xu et al., 2002; Solano et al., 2011; Li et al., 2014). Biological methods usually have advantages over chemical methods for the production of optically pure *L*-Tle (Menzel et al., 2004; Zhu L. et al., 2016). The biological methods have better enantiomeric purity, specificity of product, low cost, and high safety, which are required in the modern industry (Gerday et al., 2000). In addition, it is more environmentally friendly than chemical methods and can efficiently synthesize *L*-Tle with high optical purity (Liu et al., 2014; Ouyang et al., 2020).

Leucine dehydrogenase (EC 1.4.1.9) is an oxidoreductase enzyme that depends on the NAD^+^. It can reversibly catalyze the oxidative deamination of some aliphatic amino acids and the asymmetric reductive amination of keto acids, which is a biological pathway for the synthesis of amino acids and keto acids (Zhu W. et al., 2016; Xu et al., 2017). Leucine dehydrogenase is one of the most widely used key enzymes in the preparation of *L*-Tle by biologically catalyzed asymmetric reduction. With the development of genetic engineering and protein engineering technology, more and more leucine dehydrogenases have been expressed and identified. Li et al. (2009) have expressed and identified the leucine dehydrogenase from *Bacillus sphaericus* ATCC4525, indicating that the enzyme can asymmetrically convert corresponding α-keto acids to α-amino acid. Zhao et al. (2012) have screened leucine dehydrogenases from six psychrophilic bacteria and found that the leucine dehydrogenase from *Sporosarcina psychrophila* DSM 3 had the highest activity, the enzyme was purified to homogeneity and characterized. Li et al. (2014) have discovered a leucine dehydrogenase (*Es*LeuDH) from *Exiguobacterium sibiricum* through genome mining technology and achieved the production of *L*-Tle by coexpression of *Es*LeuDH and *Bacillus megaterium* glucose dehydrogenase (*Bm*GDH). Zhu W. et al. (2016) also have discovered a leucine dehydrogenase with excellent thermostability from *Laceyella sacchari* (*Ls*LeuDH), with a specific activity of 183 U mg^–1^ at pH 10.5 and 25°C. Ouyang et al. (2020) reported LeuDH from *Bacillus coagulans* NL01 (*Bc*LeuDH) and found that *Bc*LeuDH has excellent thermal stability and pH stability, and its thermal stability is related to hydrophobic amino acid residues and hydrophobic patches. The process of preparing *L*-Tle by the asymmetric reduction reaction of leucine dehydrogenase requires the participation of coenzymes. Coenzymes are expensive. Adding additional coenzymes will greatly increase production costs. Formate dehydrogenases (FDHs) are a group of heterogeneous enzymes found in prokaryotes and eukaryotes, which can catalyze the oxidation of formic acid to CO_2_ and H^+^ (Jormakka et al., 2003). Its catalytic substrate is sodium formate, which is cheap, and when driving the coenzyme cycle, it does not produce by-products. Considering the high enantioselectivity of leucine dehydrogenase (EC 1.4.1.9) (Baker et al., 1995; Kragl et al., 1996; Krix et al., 1997) and the continuous regeneration of NADH by formate dehydrogenase, coupling the two to form a coenzyme regeneration system is a very economical and effective method for converting trimethylpyruvic acid (TMP) into *L*-Tle.

**SCHEME 1:**
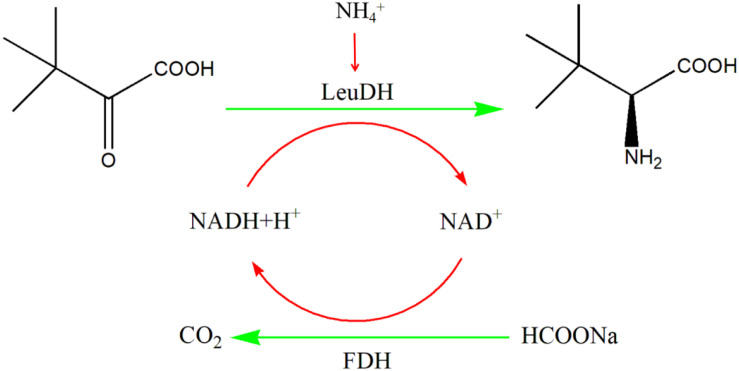
One-pot synthesis of *L*-Tle from TMP *via* biocatalysis with recombinant *E. coli* cells containing the LeuDH and FDH.

In this work, we mined five potential genes encoding leucine dehydrogenase through genome mining technology, and four genes successfully achieved soluble expression in *E. coli*. These recombinant leucine dehydrogenases were purified to homogeneity and characterized. Among them, the specific activity of leucine dehydrogenase from *Planifilum fimeticola* are also significantly better than those of other enzymes, which has great application potential. In addition, using whole cells as a biocatalyst, the formate dehydrogenase (*Cb*FDH) from *Candida boidinii* (Slusarczyk et al., 2000; Zheng et al., 2016) and *Pf*LeuDH are coexpressed to achieve the regeneration of reducing coenzyme (NADH). As shown in Scheme 1, the process of preparing *L*-Tle by whole-cell catalysis of TMP was preliminarily studied, which laid a solid foundation for the realization of low-cost and large-scale biosynthesis of *L*-Tle. More interestingly, in our study, the gene copy number of formate dehydrogenase was increased by double plasmid system, which can improve the efficiency of coenzyme regeneration and the conversion efficiency of the main reaction, thus reducing the production cost and hopefully realizing large-scale production biosynthesis of *L*-Tle.

## Materials and Methods

### Reagents and Kits

PrimeSTAR^®^ HS (Premix), DNA Marker, Restriction enzymes, and DNA Ligation Kit Ver.2.1 were purchased from TaKaRa (Dalian, China). *Bam*HI, *Hin*dIII, *Nde*I, and *Xho*I were purchased from New England Biolabs, Inc. (Ipswich, MA, England). *L*-Leucine, NAD^+^/NADH standard products were purchased from Solarbao (Beijing, China). Trimethylpyruvic acid, *L*-tert-leucine, *D*-tert-leucine, and 2,3,4,6-tetra-*O*-acetyl-β-*D*-glucopyranose isothiocyanate (GITC) were purchased from Macklin (Shanghai, China). Chromatographically, pure methanol and acetonitrile were purchased from Komiou (Tianjin, China). BCA-200 protein assay kit, IPTG, and protein markers were purchased from Sangon (Shanghai, China). Axygen^®^ AxyPrep^TM^ PCR Clean-Up Kit, Axygen^®^ AxyPrep^TM^ DNA Gel Extraction Kit, and Axygen^®^ AxyPrep^TM^ Plasmid Miniprep Kit were purchased from Corning (New York, United States).

### Strains, Plasmids, and Culture Media

*Escherichia coli* BL21(DE3), *E. coli* BL21(DE3)/pACYCDuet-1, *E. coli* BL21(DE3)/pET28a, and *E. coli* BL21(DE3)/pET28a-*Cb*FDH (Slusarczyk et al., 2000; Zheng et al., 2016) preserved by our Lab were cultured in LB medium (Tang et al., 2020a). *E. coli* BL21(DE3)/pET28a-*Pr*LeuDH, *E. coli* BL21(DE3)/pET28a-*Tb*LeuDH, *E. coli* BL21(DE3)/pET28a-*Pf*LeuDH, *E. coli* BL21(DE3)/pET28a-*Cs*LeuDH, and *E. coli* BL21(DE3)/pET28a-*Bb*LeuDH, respectively, contained leucine dehydrogenase encoding gene from *Paenibacillus riograndensis* (GenBank accession number: WP_039790706.1), *Thermoactinomycetaceae bacterium SCSIO 07575* (GenBank accession number: WP_124727229.1), *Planifilum fimeticola* (GenBank accession number: WP_106345646.1), *Caldanaerobacter subterraneus* subsp. *yonseiensis KB-1* (GenBank accession number: WP_022587968.1), and *Bdellovibrio bacteriovorus* (GenBank accession number: WP_088565218.1), which were artificially synthesized and constructed by Synbio Technologies Genes for Life after codon optimization.

### Genome Mining for Putative Leucine Dehydrogenase

**FIGURE 1:**
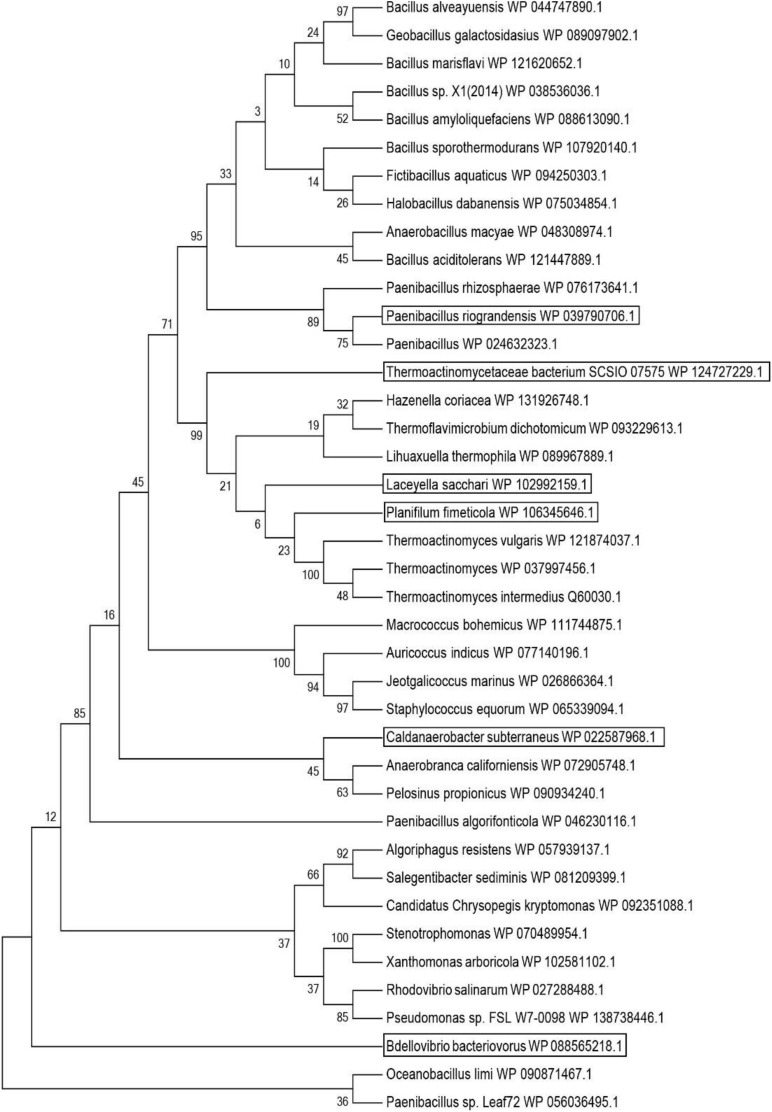
Phylogenetic tree of probe and putative leucine dehydrogenase. The tree was constructed using the MEGA 6 program with the Maximum Likelihood method.

*Ls*LeuDH is a leucine dehydrogenase with high specific activity and thermal stability encoded by *Laceyella sacchari* (GenBank accession number: WP_102992159.1) (Zhu W. et al., 2016). It is used as a probe for BLAST to search for potentially new leucine dehydrogenases. These potential leucine dehydrogenase genes mainly come from the bacterial genome sequence information and are unidentified sequences. According to the results of BLAST analysis, 10 potential leucine dehydrogenase protein sequences were respectively selected from the results of the identity interval of 100–80%, 80–70%, 70–60%, and 60–50% (Probe included). The phylogenetic tree of potentially new leucine dehydrogenases was constructed using both the ClustalX2 program and MEGA 6.0 software (Figure 1). According to the phylogenetic tree analysis, *Tb*LeuDH derived from *Thermoactinomycetaceae bacterium SCSIO 07575* (84.34% identity), *Pf*LeuDH derived from *Planifilum fimeticola* (79.28% identity), *Pr*LeuDH derived from *Paenibacillus riograndensis* (68.13% identity), *Cs*LeuDH derived from *Caldanaerobacter subterraneus* subsp. *yonseiensis KB-1* (63.87% identity), and *Bb*LeuDH derived from *Badellovabrio bacteriovorus* (58.33% identity) were selected.

### Gene Cloning and Expression of Selected Leucine Dehydrogenase

Based on the results of genome mining, the selected potential leucine dehydrogenase genes were codon optimized and artificially synthesized by Synbio Technologies Genes for Life (Suzhou, Jiangsu, China). According to this method (Tang et al., 2020a), the recombinant strains were cultured, induced, collected, and lysed by ultrasonication. The lysate supernatant was purified to electrophoretic purity by Ni-chelation affinity chromatography (Ma et al., 2009; Tang et al., 2018a,b) and centrifuged at 4°C and 8,000 rpm for 5 min to collect the induced bacterial cells. The collected cells were resuspended in 5 ml of ice-bath binding solution, then 25 μl of PMSF (10 mg ml^–1^) solution was added, followed by ultrasonication of lyse cells on ice. The collected lysis supernatant is added to a well-balanced chromatography column containing Ni-sepharose medium. To bind the target protein fully to the column, repeatedly hang the column three times. Then, 100 ml of binding solution (20 mM pH 7.9 Tri-HCl, 10 mM imidazole solution/40 mM imidazole solution/60 mM imidazole solution, 500 mM NaCl solution) and an appropriate amount of fresh eluent (20 mM pH 7.9 Tri-HCl, 200 mM imidazole solution, 500 mM NaCl solution) were used to wash the contaminated and target proteins on the column, respectively. The penetrating fluid containing the target protein is collected and saved. It is concentrated and imidazole was removed by ultrafiltration.

### Enzyme Activity and Protein Assays

Enzyme assays regarding their activities were carried out on a spectrophotometer by detecting the change of NADH absorbance (ε = 6,220 M^–1^ cm^–1^) at 340 nm for reactions of reductive amination (Luo et al., 2020). One milliliter reaction mixture contained 0.1 mM NADH, 2 mM TMP, and 500 mM NH_4_Cl-NH_3_⋅H_2_O buffer (pH 9.5). After incubation at 30°C for 2 min, 100 μl of enzyme solution diluted to an appropriate concentration was added for detecting the change in absorbance at 340 nm. The oxidative activity of formate dehydrogenase is measured by the increase of NADH by the same way as above. One milliliter reaction mixture contained 1 mM NAD^+^, 6 mM sodium formate, and 100 mM phosphate buffer (pH 7.5). According to this method (Tang et al., 2018b), the protein concentration and SDS-PAGE were determined. Then the apparent molecular weight of subunit was estimated with the Quantity One software based on the standard marker proteins (Tang et al., 2016).

### Biochemical Characterization of Purified Recombinant Enzymes

The temperature and pH characteristics of recombinant leucine dehydrogenases were measured with slight modification compared with the reported method (Tang et al., 2018b). The temperature optima of these recombinant leucine dehydrogenases were determined under the standard assay conditions described above, except for temperatures ranging from 25 to 75°C with an interval of 5°C. To estimate their thermostability, these recombinant leucine dehydrogenases were incubated at pH 9.5 and various temperatures (25–75°C) for 1.0 h, and then, the residual enzyme activity was measured under the optimal reaction temperatures. Here, thermostability was defined as a temperature, at or below which the residual activity retained more than 85% of its original activity. The pH optima of these recombinant leucine dehydrogenases were assayed by the standard activity assay method as stated above with 500 mM NH_4_Cl-NH_3_⋅H_2_O buffer over the pH range of 7.5–11.0 with an interval of 0.5. To estimate the pH stability, aliquots of these recombinant LeuDHs were preincubated at 0°C for 1.0 h in varied pH values from 7.5 to 11.0, and the residual activities were assayed under the optimal reaction temperatures and pH values. The pH stability, in this work, was defined as the pH range over which the residual activities were more than 85% of the original activity. The kinetic parameters of recombinant leucine dehydrogenase were determined according to the reported method with slight modifications (Li et al., 2014). The kinetic parameters of the purified LeuDH on the natural substrate *L*-leucine were determined by measuring activities under varied concentrations (0.1–3 mM) at a fixed NAD^+^ concentration (1 mM), and the correlation curve of reaction rate to substrate concentration was plotted and analyzed using non-linear fitting using the Origin 2018 software. In the same way, to determine the *K*_m_ and *V*_max_ for TMP, the concentration of TMP was varied from 1 to 20 mM in the presence of 0.3 mM NADH. The *K*_m_ value of LeuDH toward NADH (0.05–0.35 mM) and NAD (0.1–4 mM) was assayed as described above at a constant concentration of TMP (40 mM) and *L*-leucine (4 mM), respectively.

### Substrate Specificity of Recombinant Leucine Dehydrogenase

The 1-ml reaction mixture contains 100 mM pH 9.5 glycine-NaOH buffer, 2 mM different amino acids as substrates, 0.1 mM NAD^+^, incubated at 30°C for 2 min, and then the pure enzyme diluted with appropriate concentration was added to detect the change in absorbance at 340 nm. The enzyme activity of leucine dehydrogenase to different amino acids was measured, and the enzyme activity was taken as 100% toward 2 mM *L*-leucine. In the same way, the 1-ml reaction mixture contains 500 mM pH 9.5 NH_4_Cl-NH_3_⋅H_2_O buffer, 2 mM different keto acids as substrates, and 0.1 mM NADH to determine the enzyme activity of leucine dehydrogenase to different keto acids; the enzyme activity is taken as 100% toward 2 mM TMP.

### Construction of Recombinant *E. coli* Strains

Using pET28a-*Cb*FDH plasmid as a template to amplify the target gene containing *Bam*HI and *Hin*dIII restriction sites (McsF1: CGGGATCCGATGAAAATCGTTCTGGTT; McsR1: CCAAGCTTTTATTTTTTATCATGTTTA; PCR amplification conditions: 98°C, 5 min; 98°C, 10 s; 59°C, 15 s; 72°C, 60 s; 30 cycles; 72°C, 10 min, 4°C storage). The target gene and pACYCDuet-1 were digested with restriction enzymes *Bam*HI and *Hin*dIII, and then the digested products were purified. DNA Ligation Kit Ver.2.1 was used to connect the target gene to the vector, and the resulting plasmid was introduced into *E. coli* BL21(DE3). After chloramphenicol resistance screening, colony PCR detection, double enzyme digestion, and sequencing identification were carried out, and then the *E. coli* BL21(DE3)/pACYCDuet-1-*Cb*FDH recombinant strain was obtained. In the same way, pET28a-*Pf*LeuDH plasmid was used as a template to amplify the target gene containing *Nde*I and *Xho*I restriction sites (McsF2:CGCCATATGAAATGGTTCGACTACA; McsR2:CCG CTCGAGTTAAACCATAGTAGTA; PCR amplification conditions: 98°C, 5 min; 98°C, 10 s; 58°C, 15 s; 72°C, 60 s; 30 cycles; 72°C, 10 min, 4°C storage). The target gene and pACYCDuet-1-*Cb*FDH were digested with restriction enzymes *Nde*I and *Xho*I, and then the digested products were purified. DNA Ligation Kit Ver.2.1 was used to connect the target gene to the vector, and the resulting plasmid was introduced into *E. coli* BL21(DE3). According to the same method as above, the *E. coli* BL21(DE3)/pACYCDuet-1-*Cb*FDH-*Pf*LeuDH recombinant strain was obtained through screening, detection, and identification. Finally, the pET28a-*Cb*FDH and pACYCDuet-1-*Cb*FDH-*Pf*LeuDH plasmids were transformed into *E. coli* BL21(DE3)-competent cells, which were screened for dual resistance to kanamycin and chloramphenicol, and then the recombinant *E. coli* BL21(DE3)/pACYCDuet-1-*Cb*FDH-*Pf*LeuDH:pET28a-*Cb*FDH was obtained.

### “One-Pot” Biosynthesis of *L*-Tle From TMP

The biocatalytic reaction was carried out at 10 ml scale in 50 ml flask employing freeze-dried whole cells as catalyst. The reaction mixture contains 500 mM NH_4_Cl-NH_3_⋅H_2_O buffer (pH 9.5), 100 mM TMP, 200 mM sodium formate, and 0.1 g freeze-dried whole cells. The reaction mixture was incubated at 30°C and 200 rpm for 35 h, and 100 μl samples were periodically removed for high-performance liquid chromatography (HPLC) analysis (Fan et al., 2015; Tang et al., 2020b).

### Analysis of *L*-Tle and Biotransformation Products

The product was quantified by column derivatization method. 2,3,4,6-Tetra-*O*-acetyl-β-*D*-glucopyranose isothiocyanate (GITC) was used as a derivatization reagent to derivatize *L*-tert-leucine and *D*-tert-leucine. Chromatographic conditions: Thermo Hypersil BDS C 18 column (250 mm × 4.6 mm, 5 μm), detection wavelength: 254 nm; injection volume: 10 μl, mobile phase: V (methanol):V (phosphate) = 58:42 buffer; flow rate: 0.6 ml min^–1^. Two hundred-microliter sample and 200-μl 10-mM triethylamine solution were placed into a 1.5-ml Eppendorf tube, mixed well, added 200-μl 4-mM GITC solution, vortexed to mix, in 35°C water bath to react 60 min. Dissolved samples and reagents are prepared with V (acetonitrile):V (water) = 1:1 solution. The 10-mM *L*-Tle standard solution was diluted to 0.2, 0.4, 0.5, 0.6, 0.8, and 1.0 mM. After derivatization, it was determined by HPLC, and the peak area (*X*) was used to perform linear regression on the sample molar concentration (*Y*) to obtain the regression equation: *Y* = 0.0008*x* + 0.0172, *R*^2^ = 0.9990, and the linear range is 0.2–1.0 mM. The qualitative analysis of the reaction mixture is by UPLC-MS method at Xevo G2-XS QTof. Chromatographic conditions include the following: Poroshell 120 EC-C 18, column 2.7 μm, 3.0 × 150 mm; mobile phase: water (A, 0.1% formic acid) and acetonitrile (B, 0.1% formic acid); elution gradient program: A:B = 95:5, 5 min; A:B = 1:99, 20 min; A:B = 1:99, 24 min; A:B = 95:5, 25 min; flow rate: 0.4 ml min^–1^; injection volume: 1 μl; and column temperature: 40°C. Mass spectrometry conditions are as follows: source: electrospray ion (ESI); source temperature: 120°C; mass spectrometry scan mode: negative ion; scan range: 50–1200 m/e; capillary voltage: 2.73–2.75 kV; cone voltage: 40 V; cone gas flow: 50 L h^–1^; desolvation gas flow: 600 L h^–1^; and desolvation temperature: 450°C.

## Results and Discussion

### Gene Cloning and Expression of Leucine Dehydrogenase

**FIGURE 2:**
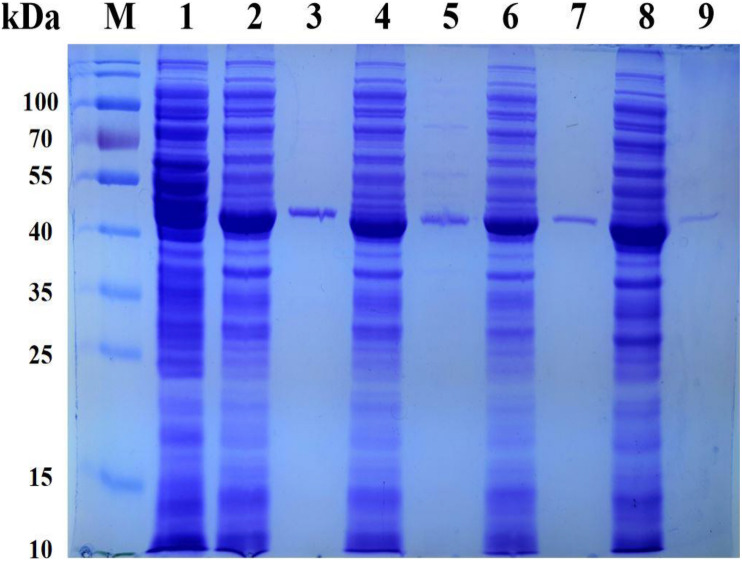
The SDS-PAGE analysis for the representative recombinant leucine dehydrogenase. Lane M, PageRuler Prestained Protein Ladder; lane 1, expressed products of *E. coli* BL21(DE3)/pET28a; lane 2, crude *Tb*LeuDH; lane 3, purified *Tb*LeuDH; lane 4, crude *Pf*LeuDH; lane 5, purified *Pf*LeuDH; lane 6, crude *Bb*LeuDH; lane 7, purified *Bb*LeuDH; lane 8, crude *Cs*LeuDH; lane 9, purified *Cs*LeuDH.

In order to efficiently biosynthesize *L*-Tle, we used genome mining strategy to find leucine dehydrogenase that can be used in the reductive amination reaction of TMP. Five gene fragments were artificially synthesized. Five potential NAD^+^-dependent leucine dehydrogenases were cloned, four of which were solublely expressed in *E. coli* BL21(DE3). One of the leucine dehydrogenases has no soluble expression; we consider that the reason for the formation of inclusion bodies by this leucine dehydrogenase may be caused by excessive and rapid expression. It is also possible that the presence of the secretory sequence of the recombinant protein hinders folding, leading to the production of misfolded molecules. All four soluble leucine dehydrogenases have catalytic activity for TMP. In order to characterize its enzymatic properties, the recombinant leucine dehydrogenase was purified by Ni-NTA affinity chromatography. The SDS-PAGE analysis of four recombinant leucine dehydrogenases is shown in Figure 2. As shown in Figure 2, the purified protein has reached electrophoretic purity with an apparent molecular weight of 40.0 kDa. The specific activity of purified *Pf*LeuDH, *Tb*LeuDH, *Bb*LeuDH, and *Cs*LeuDH is 51.02, 24.18, 13.28, and 1.75 U mg^–1^, respectively. Among them, the leucine dehydrogenase from *Planifilum fimeticola* (GenBank accession number: WP_106345646.1), named as *Pf*LeuDH in this paper, has the highest activity (582.64 U ml^–1^ under optimal temperature). The amino acid sequence of *Pf*LeuDH is 79.28% identical to that of the probe, indicating that it is a new type of leucine dehydrogenase. Using BLASTp in the NCBI protein database, *Pf*LeuDH was found to belong to the Glu/Leu/Phe/Val dehydrogenase family.

### Comparative Enzymatic Properties of Leucine Dehydrogenase

**FIGURE 3:**
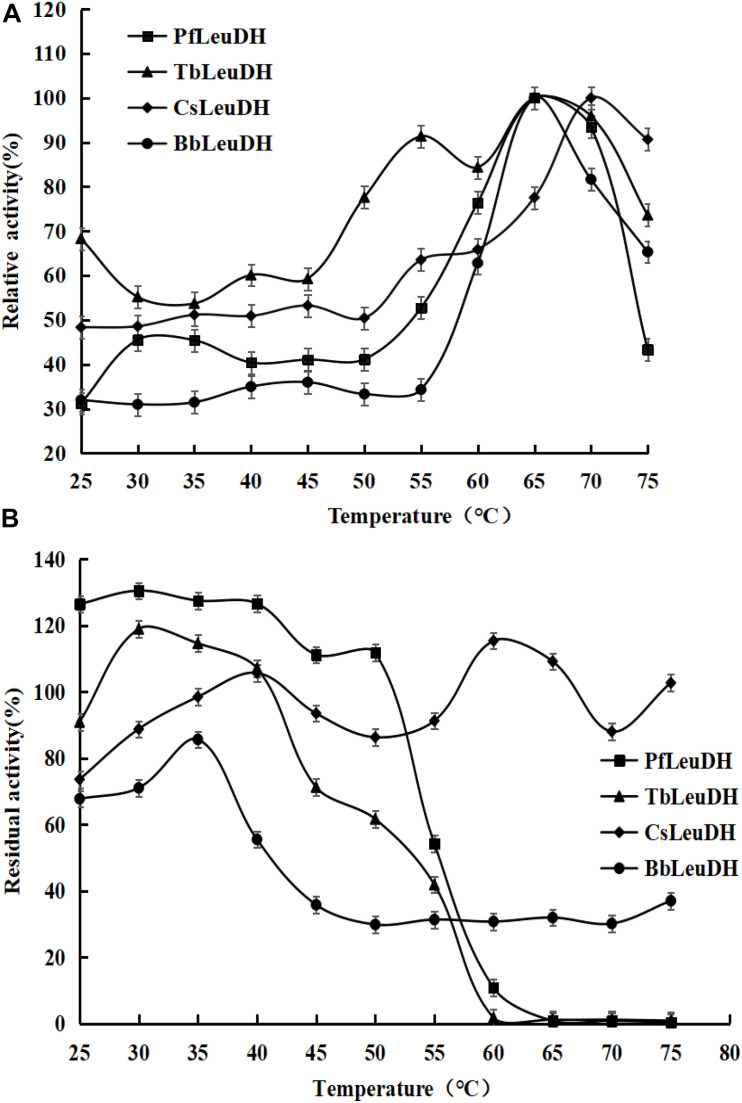
The temperature properties of the representative recombinant leucine dehydrogenases. **(A)** Temperature optima of the representative recombinant leucine dehydrogenases. The temperature optima were assayed spectrophotometrically by monitoring the increase of NADH absorbance at 340 nm at reaction temperatures ranging from 25 to 75°C. **(B)** Temperature stabilities of the representative recombinant leucine dehydrogenases. The temperature stabilities were determined by incubating them at various temperatures (25–75°C) for 1.0 h, and then the residual enzyme activities were measured as describe in the text.

**FIGURE 4:**
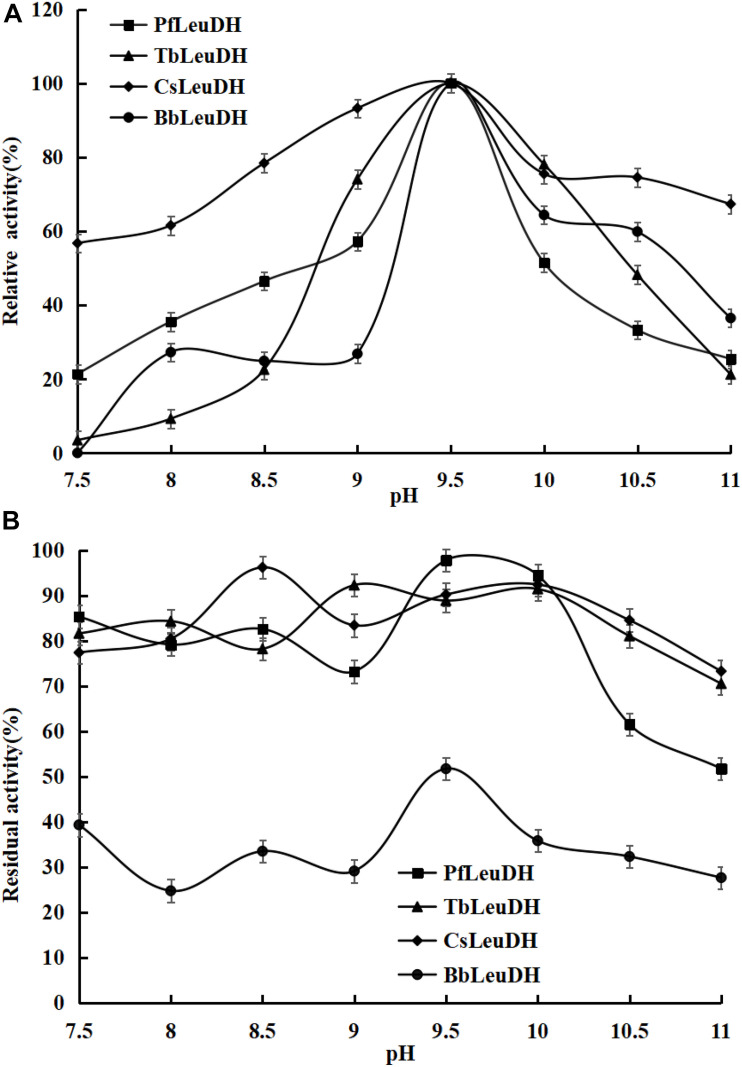
The pH properties of the representative recombinant leucine dehydrogenases. **(A)** pH optima of the representative recombinant leucine dehydrogenases. The pH optima were assayed by the standard activity assay method as described in the text with 500 mM NH_4_Cl-NH_3_⋅H_2_O buffer over the pH range of 7.5-11.0. **(B)** pH stabilities of the representative recombinant leucine dehydrogenases. To estimate the pH stability, aliquots of these recombinant leucine dehydrogenase solutions were preincubated at 0°C for 1.0 h at various pH values from 7.5 to 11.0, and the residual activities were assayed under the optimal reaction temperatures and pH values.

In order to compare the enzymatic properties of purified recombinant leucine dehydrogenases, the 2-oxo-4-methylpentanoic acid was used as the model substrate to determine the corresponding parameters. The temperature properties of these purified leucine dehydrogenases are shown in Figure 3, the temperature optimum of *Pf*LeuDH, *Tb*LeuDH, and *Bb*LeuDH was 65°C, while the one of *Cs*LeuDH was 70°C (Figure 3A). Ouyang et al. (2020) have shown that the optimum temperature of *Bc*LeuDH from *Bacillus coagulans NL01* was 50°C. In addition, a thermotolerant *L*-leucine dehydrogenase from *Laceyella sacchari* with an optimal temperature of 60°C was reported by Zhu W. et al. (2016). The optimal temperature of these leucine dehydrogenases is greater than the optimal physiological temperature of *E. coli*. The purified *Pf*LeuDH was highly stable at below 50°C after being incubated for 1 h, and their residual activities were over 100% (Figure 3B). It shows that the enzyme has good thermal stability below 50°C. For this phenomenon, we speculate that the low-temperature treatment may activate the enzyme with slight changes in its structure. Unfortunately, when the temperature is greater than 50°C at each temperature for 1 h, the residual enzyme activity of *Pf*LeuDH is less than 60%. Hence, to eliminate the influence of bad thermostability, whole cell catalysis was preferred at 30°C in reductive amination reaction of TMP. The pH properties of the purified leucine dehydrogenases are shown in Figure 4. As shown in Figure 4A, all of them are alkalophilic dehydrogenases, and the pH optimum of all LeuDHs was 9.5. *Cs*LeuDH has the widest pH adaptability and can maintain more than 80% of its activity between 8 and 10.5. At pH 9–10, *Pf*LeuDH and *Tb*LeuDH can maintain more than 85% of their original activity (Figure 4B). They displayed extreme alkaline pH for the conversion of formate, which was consistent with the *Mt*FDH from *Myceliophthora thermophile* (Altaş et al., 2017). However, there was a significant difference compared with the *Bb*FDH come from *Burkholderia dolosa* PC543 (Alpdagtas et al., 2018).

The kinetic parameters of the purified *Pf*LeuDH on TMP and NADH, as well as leucine and NAD^+^ are determined and summarized in Table 1. Table 1 compares the enzymatic parameters of *Pf*LeuDH with other reported LeuDHs in Table 2. According to Tables 1, 2, the *K*_*m*_ value of *Pf*LeuDH for TMP was 3.08 mM, which was lower than the reported LeuDHs, indicating that *Pf*LeuDH has a higher affinity for TMP. In addition, the *K*_cat_/*K*_m_ value of *Pf*LeuDH was 12.69 s^–1^ mM^–1^, which was distinctly higher than other reported LeuDHs, which further indicates the advantage of using *Pf*LeuDH in the biosynthesis of *L*-Tle. The considerably low *K*_m_ of *Pf*LeuDH toward *L*-leucine proved that *L*-leucine was the native substrate of LeuDH (Li et al., 2014).

**TABLE 1:** The kinetic parameter of *Pf*LeuDH.

Substrate	Kinetic parameter			
	
	K_m_	V_max_	K_cat_/	K_cat_/K_m_
	(mM)	[μmol (min mg)^–1^]	(S^–1^)	(S^–1^ mM^–1^)
Trimethylpyruvate	3.08	58.61	39.07	12.69
NADH	0.06	9.09	6.06	101
*L*-Leucine	0.1	3.49	2.33	23.3
NAD^+^	0.11	2.72	1.81	16.45

**TABLE 2:** Comparison of *Pf*LeuDH with other reported LeuDHs.

Origin	Substrate	*K*_*m*_ (mM)	*V*_max_ [μmol (min mg)^–1^]	*K*_*cat*_ (S^–1^)	*K*_*cat*_/*K*_*m*_ (S^–1^ mM^–1^)	References
*Planifilum fimeticola*	TMP	3.08	58.61	39.07	12.69	This work
*Exiguobacterium sibiricum*	TMP	5.96	67.48	45.43	7.62	Luo et al., 2020
*B. coagulans NL01*	TMP	13.20	–	21.30	1.61	Ouyang et al., 2020
*Lysinibacillus sphaericus*	TMP	12	–	31	2.6	Zhu L. et al., 2016
*Bacillus cereus*	TMP	3.75	–	0.016	0.004	Zhou et al., 2019

### Substrate Specificity of Recombinant Leucine Dehydrogenases

The catalytic reaction of leucine dehydrogenase is a reversible reaction, namely reductive amination reaction and oxidative deamination reaction (Li et al., 2009). The substrate specificity of *Pf*LeuDH for both reductive amination and oxidative deamination is investigated in Table 3. In the reductive amination reaction, *Pf*LeuDH showed the highest activity to phenylglyoxylic acid but not to pyruvic acid. The reaction that catalyzes the formation of *L*-Tle from TMP is a reversible reaction. In the oxidative deamination reaction, the lower the activity to *L*-Tle, the stronger the advantage. The relative enzyme activity of *Pf*LeuDH to *L*-Tle is only 8%. It shows that *Pf*LeuDH has great advantages in the reductive amination reaction of biosynthesis of *L*-Tle.

**TABLE 3:** Substrate specificity of *Pf*LeuDH in reductive amination and oxidative deamination.

Substrate	Relative activity (%)	Substrate	Relative activity (%)
			
	Reductive amination		Oxidative deamination
Trimethylpyruvate	100	*L*-Leucine	100
2-Oxo-4-methylpentanoic acid	235	*L*-Tert-leucine	8
Phenylglyoxylic acid	322	*L*-Phenylglycine	14
2-Oxobutyric acid	62	*L*-2-Aminobutyric acid	8
Pyruvate	0	*L*-Valine	56
		*L*-Alanine	0
		*L*-Phenylalanine	4

### Construction of Recombinant *E. coli* Strains

Recombinant *Escherichia coli* was constructed according to the method discussed previously. As shown in Supplementary Figure 1, the length of PCR and digestion products was 1,300 and 1,100 bp, respectively, which was consistent with the theoretical ones. In addition, the sequencing results showed that the reading frame and base sequence of the selected recombinant plasmid pACYCDuet-1-*Cb*FDH were exactly the same as expected. Similarly, as shown in Supplementary Figure 2, the length of PCR and digestion products was 1,300 and 1,100 bp, respectively, which was consistent with the theoretical ones. In addition, the sequencing results showed that the reading frame and base sequence of the selected recombinant plasmid pACYCDuet-1-*Cb*FDH-*Pf*LeuDH were exactly the same as expected. Finally, the pET28a-*Cb*FDH was transformed into *E. coli* BL21(DE3)/pACYCDuet-1-*Cb*FDH-*Pf*LeuDH competent cells and screened by LB plate containing chloramphenicol and kanamycin, the recombinant *E. coli* BL21(DE3)/pACYCDuet-1-*Cb*FDH-*Pf*LeuDH:pET28a-*Cb*FDH was obtained. In our study, the gene copy number of formate dehydrogenase was increased by double plasmid system, which can improve the efficiency of coenzyme regeneration and the conversion efficiency of the main reaction, thus reducing the production cost and hopefully realizing large-scale production biosynthesis of *L*-Tle.

### Expression and Analysis of Recombinant *E. coli* Strain

**FIGURE 5:**
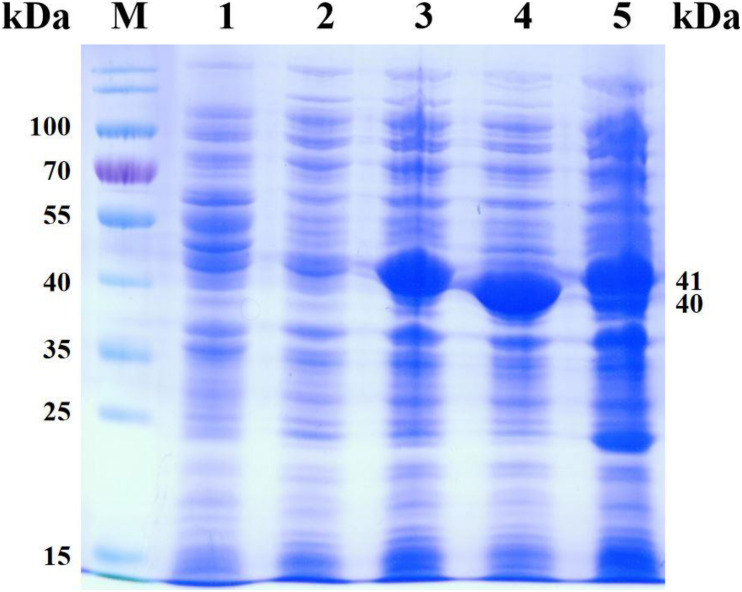
SDS-PAGE analysis for the products of co-express. Lane M, PageRuler Prestained Protein Ladder; lane 1, expression products of *E. coli* BL21(DE3)/pET28a; lane 2, expression products of *E. coli* BL21(DE3)/pACYCDuet-1; lane 3, expression products of *E. coli* BL21 (DE3)/pET28a-*Cb*FDH; lane 4, expression products of *E. coli* BL21(DE3)/pET28a-*Pf*LeuDH; lane 5, expression products of *E. coli* BL21(DE3)/pACYCDuet-1-*Cb*FDH-*Pf*LeuDH:pET28a-*Cb*FDH.

The recombinant *E. coli* BL21(DE3)/pET28a, *E. coli* BL21(DE3)/pACYCDuet-1, *E. coli* BL21(DE3)/pET28a-*Cb*FDH, *E. coli* BL21(DE3)/pET28a-*Pf*LeuDH, and *E. coli* BL21(DE3)/pACYCDuet-1-*Cb*FDH-*Pf*LeuDH:pET28a-*Cb*FDH were cultured and induced under low temperature and low concentration inducer, as described previously (Tang et al., 2019). The SDS-PAGE analysis of recombinant *E. coli* expression products is shown in Figure 5. As shown in Figure 5 (lane 5), the supersonic lysates of *E. coli* BL21(DE3)/pACYCDuet-1-*Cb*FDH-*Pf*LeuDH:pET28a-*Cb*FDH showed two specific bands with apparent molecular weights of 41 and 40 kDa, which were consistent with the theoretical ones of the *Cb*FDH and *Pf*LeuDH. These results indicated that the enzymes of *Cb*FDH and *Pf*LeuDH had been successfully expressed in the recombinant *E. coli* strain, and the recombinant enzymes were soluble.

As shown in lanes 4 and 5, the protein expression of *Pf*LeuDH is significantly reduced, which may be caused by different vectors or the effect of coexpression. Additionally, according to the reported methods (Li et al., 2014; Tang et al., 2019), the *Cb*FDH and *Pf*LeuDH activities of the supersonic lysates were 0.17 and 14.41 U ml^–1^ of fermentation liquor at 30°C, respectively. Unfortunately, the expression ratio of two enzymes was not very ideal, which still needs to be further optimized in our subsequent studies by appropriate strategy (Tang et al., 2020b). Then, the induced recombinant *E. coli* cells were collected and washed with deionized water three times and freeze-dried to dry powder (Tang et al., 2020b).

### One-Pot Biosynthesis of *L*-Tle From TMP

**FIGURE 6:**
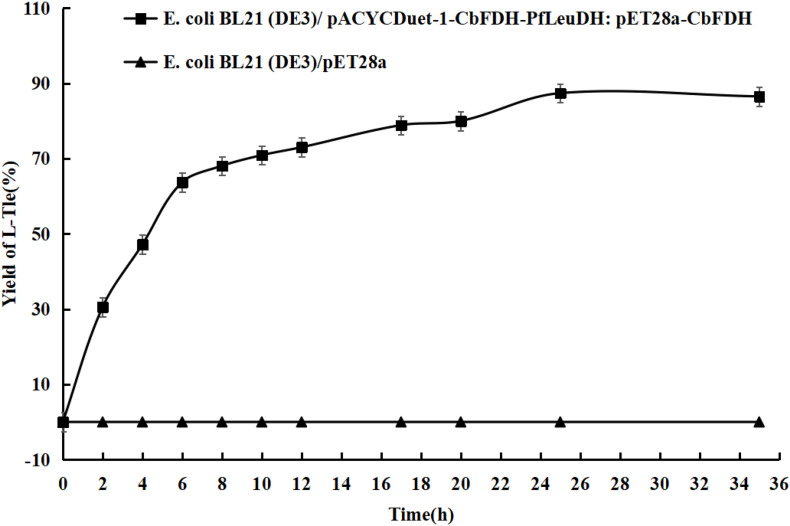
The conversion curves of TMP catalyzed by engineered *E. coli* strains. Fed-batch feeding strategy was adopted to transform 100 mM of TMP. Without adding coenzyme, the 10-ml reaction system contains 100 mM trimethylpyruvate, 200 mM sodium formate, 500 mM NH_4_Cl-NH_3_⋅H_2_O buffer, and 0.1 g freeze-dried recombinant bacterial powder. The reaction is at 30°C and 200 rpm and continued for 35 h.

**FIGURE 7:**
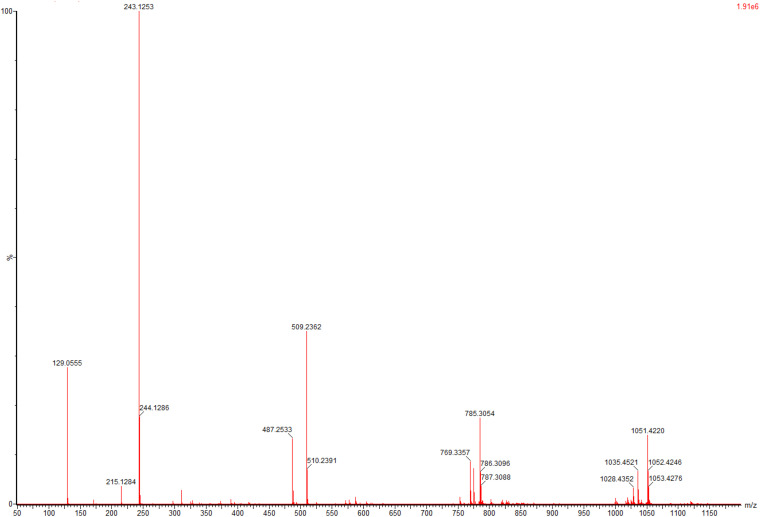
The UPLC-MS analysis of substrates in catalytic products.

**FIGURE 8:**
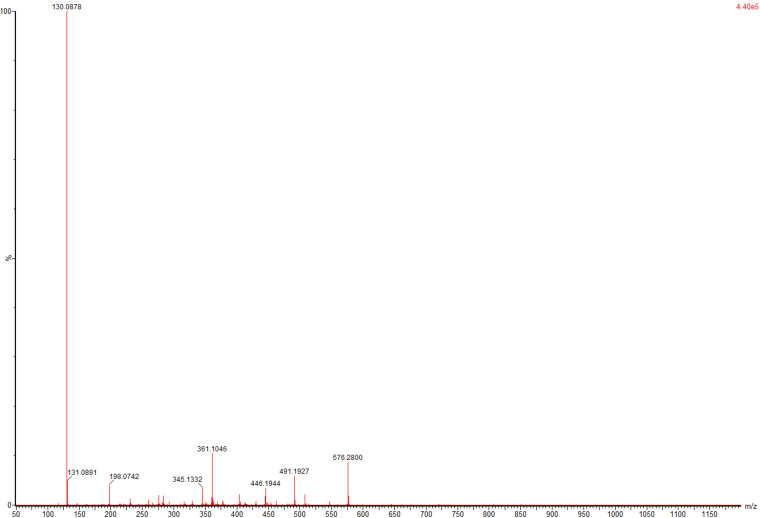
The UPLC-MS analysis of target products in catalytic products.

To evaluate application potential of the engineered *E. coli* strains in transforming TMP acid to give *L*-Tle, the one-pot biosynthesis of *L*-Tle from TMP was performed, as mentioned above. In this section, freeze-dried cells are chosen for catalytic reaction because freeze-dried cells are easier to quantify than wet cells. After inducing a large number of cells, they are freeze-dried and stored for the next use, saving time and cost. As shown in Figure 6, through a fed-batch strategy to transform 100 mM TMP, the reaction reached equilibrium at 25 h, the space-time yield of *L*-Tle was 10.90 g L^–1^ day^–1^. After 100 mM, TMP was catalyzed for 35 h by 0.1 g freeze-dried whole cells, and the reaction mixture was used for HPLC analysis as mentioned above. Ordinary HPLC analysis results with a Thermo Hypersil C 18 column showed that the reaction mixture exhibited one distinct characteristic peak with retention time of 11.24 min (Supplementary Figure 3) at above analytic condition, which was basically consistent with the 11.26 min of *L*-Tle (Supplementary Figure 4). The derivatized *D*-tert-leucine standard product has one distinct characteristic peak at about 15.52 min (Supplementary Figure 5). The result of HPLC analysis of the reaction mixture was calculated, and under the above reaction conditions, the yield and enantiomeric excess (*e.e.*) value of *L*-Tle were 87.38 and 99.99%, respectively. The UPLC-MS analysis results of the reaction mixture are shown in Figures 7, 8. In Figure 7, the mass charge ratio of the substance is 129.06. According to the mass-to-charge ratio, the molecular formula of the substance may be C_6_H_9_O_3_^–^(M-H)^–^, indicating that the substance may be C_6_H_10_O_3_ (TMP) with one H^+^ removed. In Figure 8, the mass-to-charge ratio of another substance is 130.09. According to the mass-to-charge ratio, the molecular formula of the substance may be C_6_H_12_O_2_^–^(M-H)^–^, indicating that the substance may be C_6_H_13_O_2_ (*L*-Tle) with one H^+^ removed. In addition, the mass spectrum of target product in the reaction mixture was consistent with the *L*-Tle standard product (Supplementary Figure 7).

In conclusion, using the highly active leucine dehydrogenase *Ls*LeuDH as a probe, we mined a novel leucine dehydrogenase with higher affinity and catalytic efficiency on TMP by genome mining, which was named as *Pf*LeuDH. In addition, the highly selective biosynthesis of *L*-Tle from TMP was successfully carried out by whole-cell catalysis using engineered *E. coli* cells as biocatalyst, which can efficiently coexpress leucine dehydrogenase and formate dehydrogenase. One hundred-millimolar TMP was catalyzed for 25 h, and the yield and space-time yield of *L*-Tle reached 87.38% (*e.e.* > 99.99%) and 10.90 g L^–1^ day^–1^. In short, this research has initially achieved the biosynthesis of *L*-Tle, laying a solid foundation for the realization of low-cost and large-scale biosynthesis of *L*-Tle.

## Data Availability Statement

All datasets generated for this study are included in the article/Supplementary Material, further inquiries can be directed to the corresponding author/s. Additional experimental results and other data are available free of charge *via* the Internet at http://pubs.acs.org.

## Author Contributions

Y-YJ: conceptualization and writing—original draft. H-LS: methodology and investigation. Y-LX: investigation and software. L-LY: investigation and data curation. Y-FL: funding acquisition. S-PZ: data curation and methodology. C-DT and L-GY: writing—reviewing and editing. Y-CK: funding acquisition. All authors contributed to the article and approved the submitted version.

## Conflict of Interest

The authors declare that the research was conducted in the absence of any commercial or financial relationships that could be construed as a potential conflict of interest.

## Supplementary Material

Supplementary Figure 1 The PCR and double digestion detection of recombinant *E. coli* BL21(DE3)/pACYCDuet-1-*Cb*FDH.

Supplementary Figure 2 The PCR and double digestion detection of recombinant *E. coli* BL21(DE3)/pACYCDuet-1-*Cb*FDH-*Pf*LeuDH.

Supplementary Figure 3 The HPLC analysis of reaction mixture at Thermo Hypersil C18 column.

Supplementary Figure 4 The HPLC analysis of *L*-tert-leucine at Thermo Hypersil C18 column.

Supplementary Figure 5 The HPLC analysis of *D*-tert-leucine at Thermo Hypersil C18 column.

Supplementary Figure 6 The UPLC-MS analysis of TMP.

Supplementary Figure 7 The UPLC-MS analysis of *L*-Tle.
