# Supplementary material for: Expression of Novel L-Leucine Dehydrogenase and High-Level Production of L-Tert-Leucine Catalyzed by Engineered Escherichia coli
Source: Front Bioeng Biotechnol. 2021 Mar 30;9:655522. doi: 10.3389/fbioe.2021.655522 (PMC8042219; doi:10.3389/fbioe.2021.655522)
Supplement: Supplementary Figure 1 — The PCR and double digestion detection of recombinant E. coli BL21(DE3)/pACYCDuet-1-CbFDH. [file Data_Sheet_1.docx]

**Expression of Novel Leucine Dehydrogenase and High Level Production of *L*-tert-leucine Catalyzed by Engineered *Escherichia* *coli***

**Supporting information**


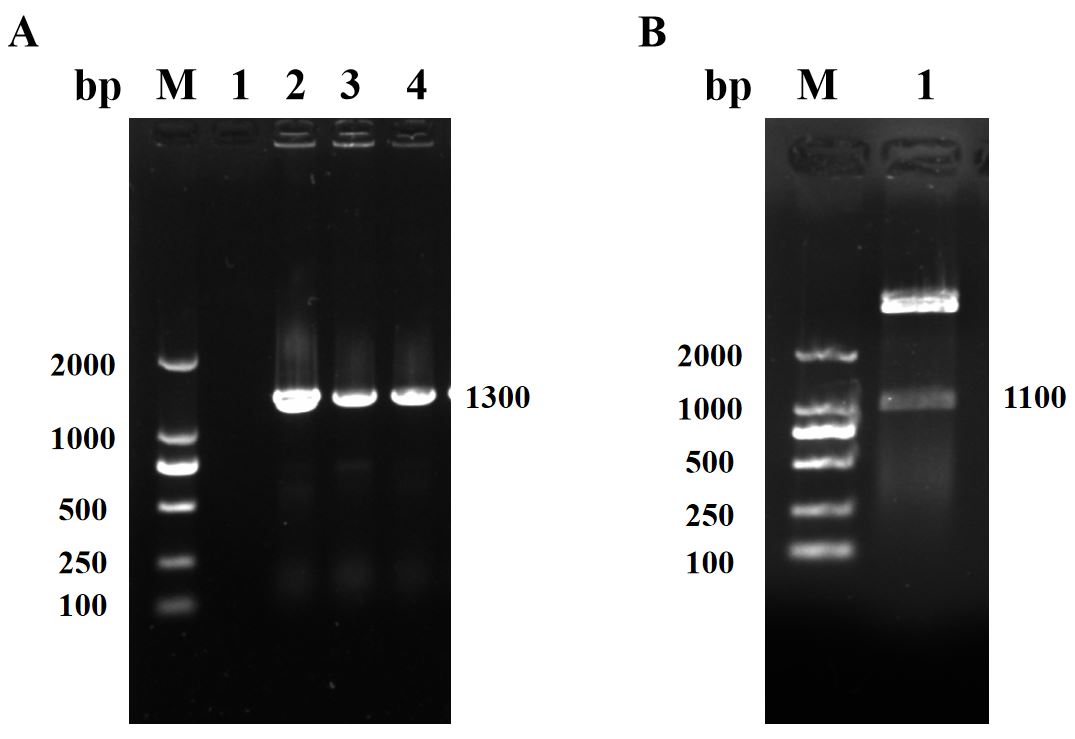


**Figure S1. The PCR and double digestion detection of recombinant *E. coli* BL21(DE3)/pACYCDuet-1-*****Cb*FDH.** (A) PCR detection. Lane M, DNA marker; lane 2-4, PCR products from different strains of *E. coli* BL21(DE3)/pACYCDuet-1-*Cb*FDH; (B) Double digestion detection. Lane M, DNA marker; lane 1, Double digestion products of pACYCDuet-1-*Cb*FDH by *Bam*H Ⅰ and *Hin*d III.

**
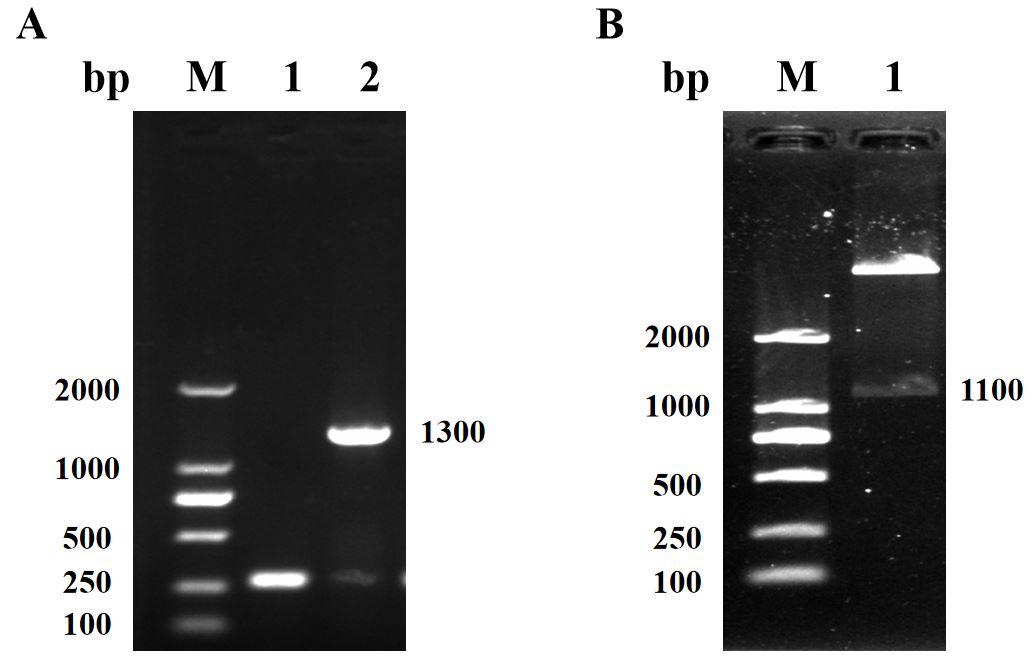
**

**Figure S2. The PCR and double digestion detection of recombinant *E. coli* BL21(DE3)/****pACYCDuet-1-*Cb*FDH-*Pf*LeuDH.** (A) PCR detection. Lane M, DNA marker; lane 1, a negative clone that has not successfully inserted the target gene; lane 2, PCR products from different strains of *E. coli* BL21(DE3)/ pACYCDuet-1-*Cb*FDH-*Pf*LeuDH; (B) Double digestion detection. lane M, DNA marker; lane 1, Double digestion products of pACYCDuet-1-*Cb*FDH-*Pf*LeuDH by *Nde* Ⅰ and *Xho* Ⅰ.


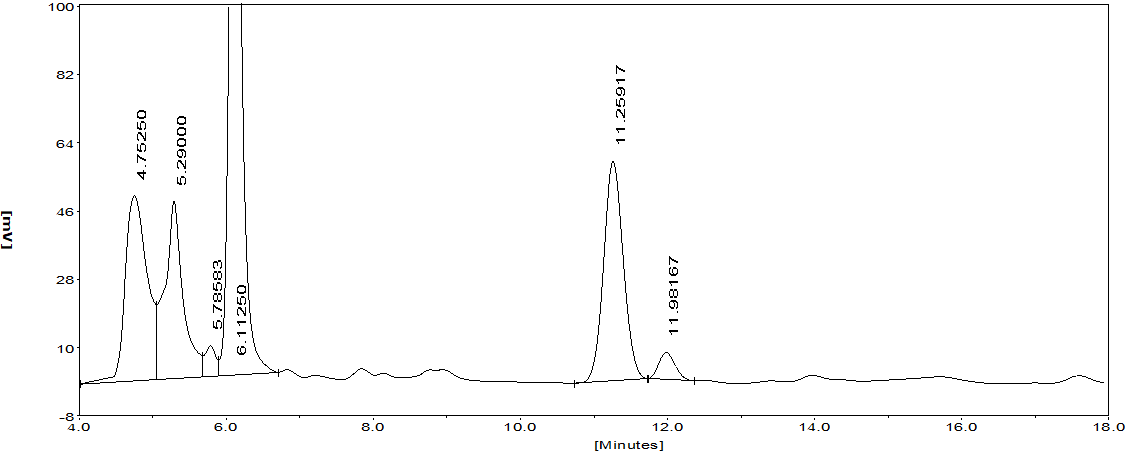


**Figure S3. The HPLC analysis of catalytic products at Thermo Hypersil C18 column.** The HPLC analysis was equipped with a Thermo Hypersil C18 column (250 mm × 4.6 mm, 5 μm, Thermo Fisher Scientific), and detected by UV detector at 254 nm. The mobile phase contained 58% methanol and 42% phosphate, and the ﬂow rate was 0.6 mL·min^−1^.


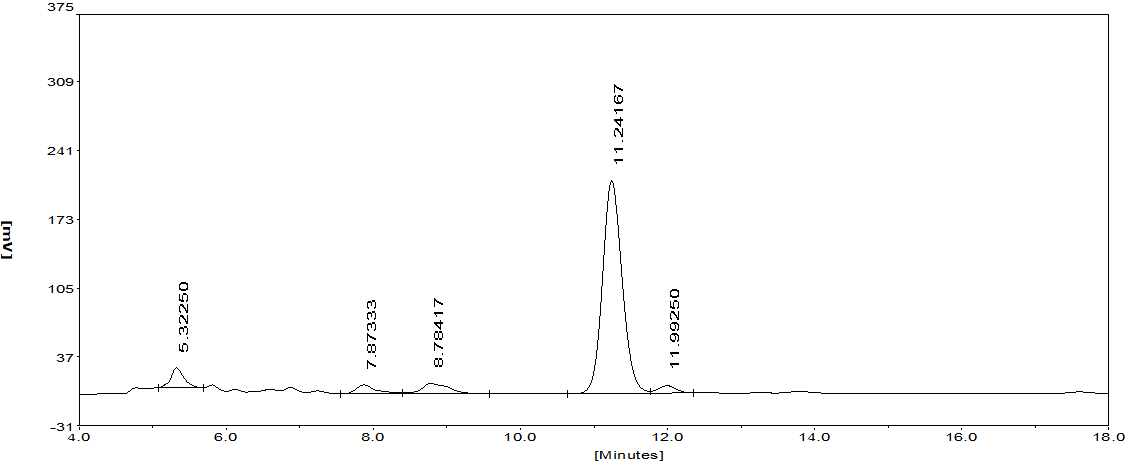


**Figure S4. The HPLC analysis of *L*-tert-leucine at Thermo Hypersil C18 column.** The HPLC analysis was equipped with a Thermo Hypersil C18 column (250 mm × 4.6 mm, 5 μm, Thermo Fisher Scientific), and detected by UV detector at 254 nm. The mobile phase contained 58% methanol and 42% phosphate, and the ﬂow rate was 0.6 mL·min^−1^.


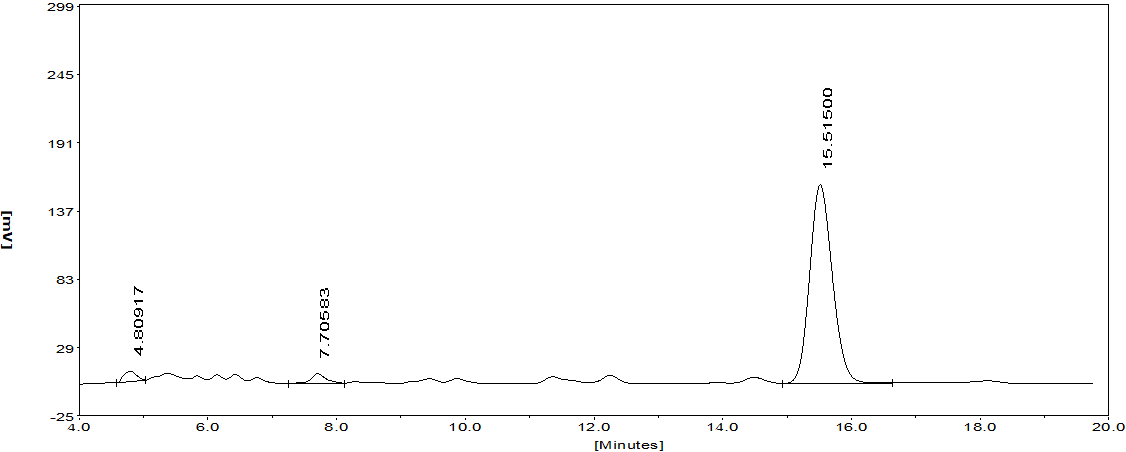


**Figure S5. The HPLC analysis of *D*-tert-leucine at Thermo Hypersil C18 column.** The HPLC analysis was equipped with a Thermo Hypersil C18 column (250 mm × 4.6 mm, 5 μm, Thermo Fisher Scientific), and detected by UV detector at 254 nm. The mobile phase contained 58% methanol and 42% phosphate, and the ﬂow rate was 0.6 mL·min^−1^.


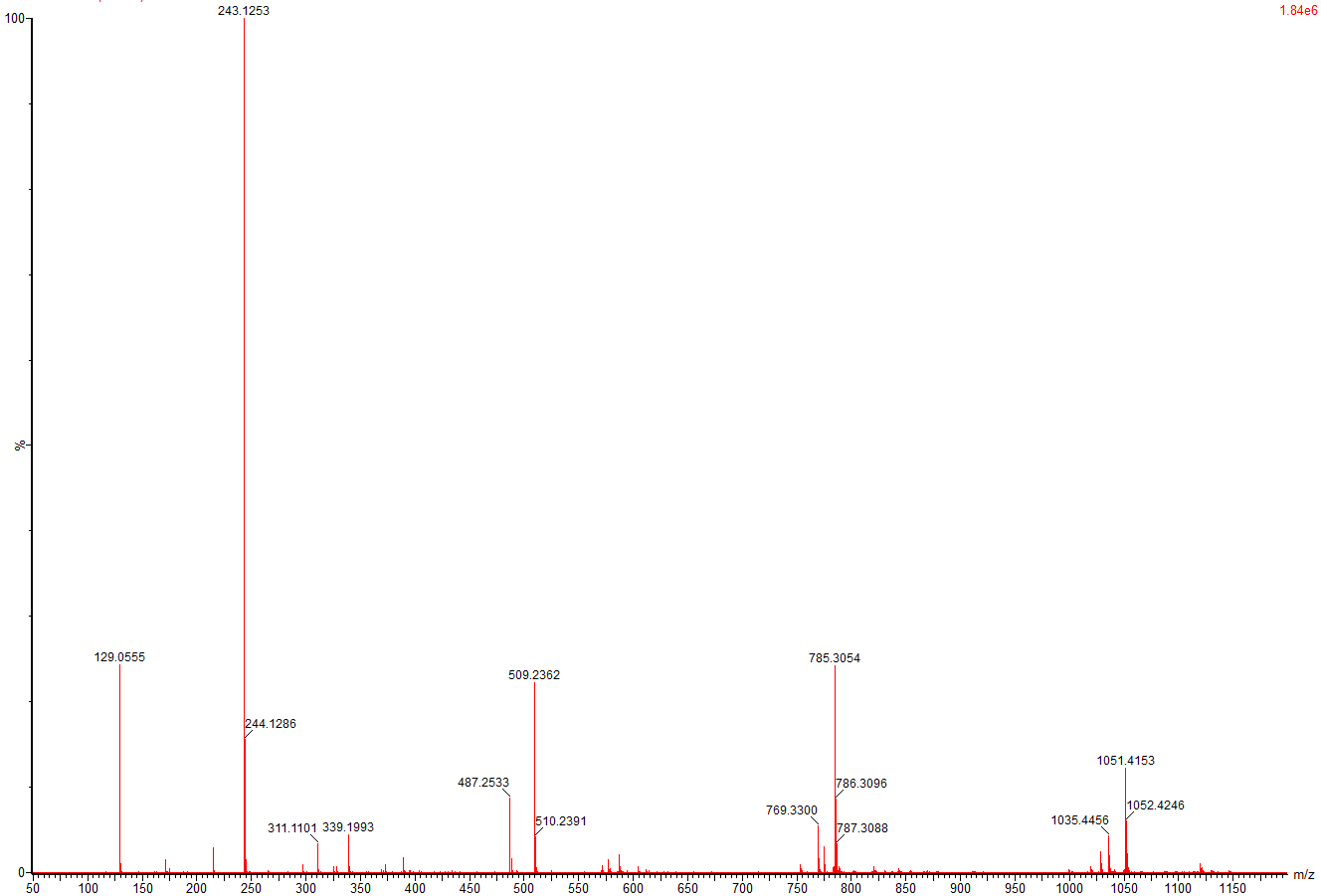


**Figure S6.** **The HRMS analysis of TMP.** The TMP was characterized by high resolution mass spectrometric analysis at Waters Xevo G2-XS QT.


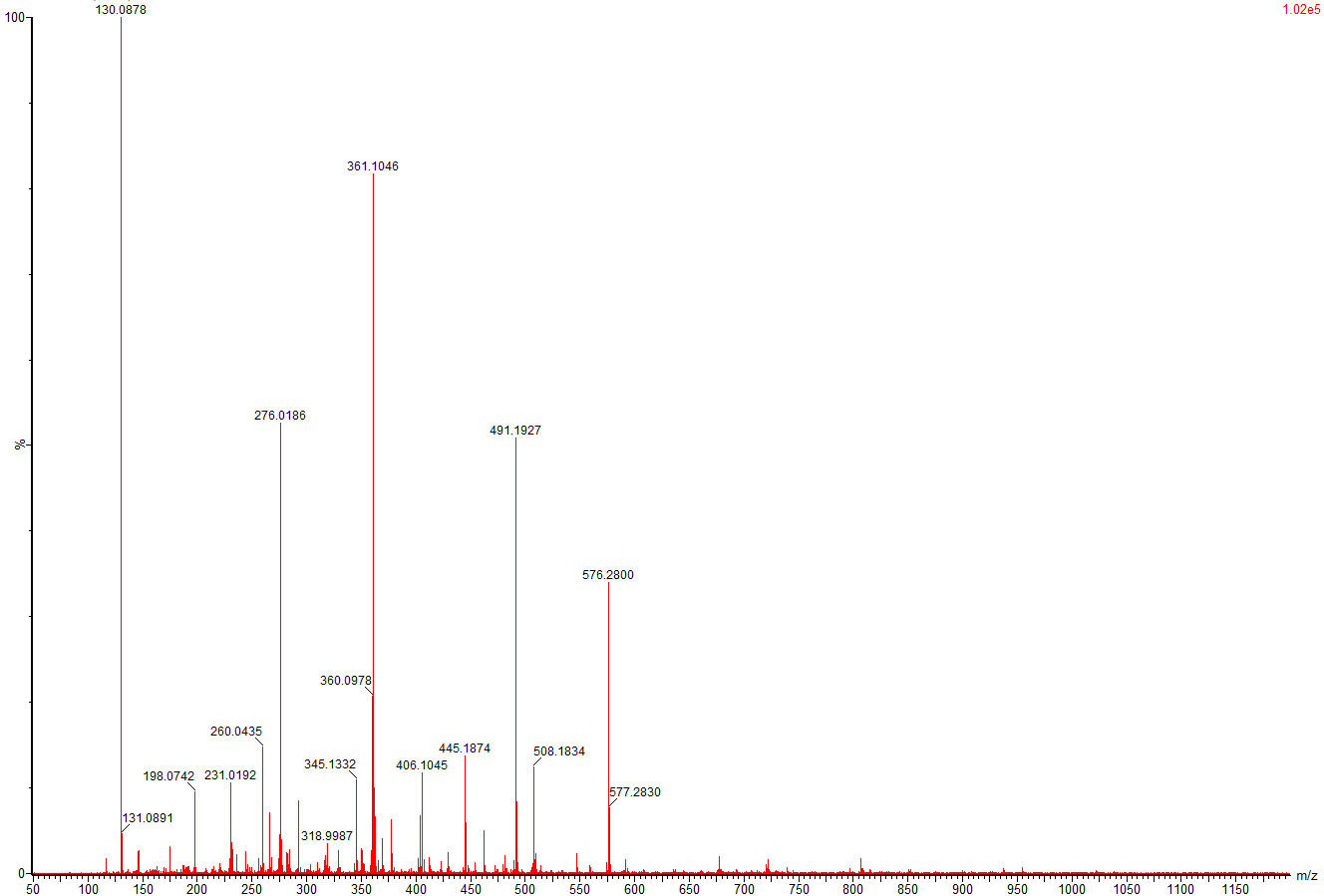


**Figure S7. The HRMS analysis of *L*-Tle.** The *L*-Tle was characterized by high resolution mass spectrometric analysis at Waters Xevo G2-XS QT**.**
